# Visual Distortions in Human Amblyopia Are Correlated with Deficits in Contrast Sensitivity

**DOI:** 10.1523/JNEUROSCI.1111-25.2025

**Published:** 2025-09-17

**Authors:** Farzaneh Olianezhad, Jianzhong Jin, Sohrab Najafian, Akihito Maruya, Qasim Zaidi, Jose-Manuel Alonso

**Affiliations:** ^1^Department of Biological and Visual Sciences, SUNY Optometry, New York, New York 10036; ^2^Department of Neurobiology, Harvard Medical School, Boston, Massachusetts 02115

**Keywords:** computational modeling, developmental disorder, LGN, shape perception, visual cortex

## Abstract

Amblyopia (lazy eye) is a developmental disorder of the visual cortex that causes deficits in visual acuity and shape perception. The loss of visual acuity is thought to originate from weakened cortical responses to stimuli. Here, we provide evidence for a similar mechanism to explain distortions in shape perception. We introduce a computational model that simulates perceptual distortions of grating patterns drawn by humans with amblyopia (Barrett et al., 2003). The model simulates a large variety of distortions by performing a weighted sum of rectified sinusoidal gratings (average, 3.3 gratings ∼6 times larger than foveal receptive fields in the primary visual cortex) with different dark/light duty cycles. The simulations accurately reproduce self-reported perceptions of amblyopic patients and decrease drawing-percept differences when ideal percepts (stimuli) are replaced with simulated percepts (9.03 ± 12.37%; *p* = 0.0002; Wilcoxon test comparing normalized Laplacian pyramid distances; Laparra et al., 2016). The simulations also reveal an increase in the number of stimulus orientations contributing to visual percepts in amblyopia and a strong correlation between contrast sensitivity deficits and both magnitude of perceived visual distortions (*r* = 0.96; *p* = 0.0007) and predicted spread of cortical activation (*r* = 0.82; *p* = 0.02). The results also demonstrate a compensatory shift in the spatial frequency distribution of cortical filters in amblyopia, which closely resembles the spatial frequency shift caused by contrast reduction in thalamocortical inputs of male cats. Taken together, our results indicate that amblyopia compensates weakened cortical responses by increasing the spread of cortical activation to include neurons with mismatched stimulus preferences that cause perceptual distortions.

## Significance Statement

Amblyopia (lazy eye) affects millions of humans worldwide, yet the neural mechanisms underlying its perceptual deficits remain poorly understood. Here, we introduce a computational model that accurately simulates a large variety of amblyopic perceptual distortions with a weighted sum of rectified sinusoidal gratings. The simulations reveal strong correlations among amblyopia deficits in contrast sensitivity, distortions in shape perception, and predicted cortical spread. Based on these results, we propose a cortical mechanism that compensates amblyopia weakened responses by increasing cortical spread to neurons with mismatched stimulus preferences that distort perception. Taken together, our results provide a mechanistic framework that links visual deficits in contrast sensitivity with distortions in shape perception while providing new insights into how developmental visual disorders alter sensory processing.

## Introduction

Amblyopia, often referred to as “lazy eye,” is a neurodevelopmental disorder of the visual brain that causes deficits in vision not correctable with lenses. Amblyopia develops early in life, as a consequence of strabismus, anisometropia, or form deprivation (Von Noorden, 1985; Ciuffreda et al., 2008), and can cause deficits in contrast sensitivity (Gstalder and Green, 1971; Hess and Howell, 1977; Hilz et al., 1977; Levi and Harwerth, 1977; Thomas, 1978; Bradley and Freeman, 1981; Movshon et al., 1987; McKee et al., 2003), binocular fusion, and depth perception (Birch, 2013; Levi et al., 2015; Kiorpes and Daw, 2018). Amblyopia can also cause distortions in shape perception. Early observations by Pugh (Pugh, 1958) described subtle but discernible perceptual distortions of high-contrast letters in amblyopic individuals, and later studies demonstrated similar distortions with local stimuli (Sireteanu et al., 1993) and sinusoidal gratings (Hess et al., 1978; Bradley and Freeman, 1985; Thibos and Bradley, 1993; Barrett et al., 2003). Notably, these visual distortions were found to vary substantially across subjects and across stimulus orientations and spatial frequencies within the same subject (Barrett et al., 2003). Collectively, these findings highlight the heterogeneous nature of perception in amblyopia.

Amblyopia affects visual function in the primary visual cortex (V1) more than in the lateral geniculate nucleus (LGN; Hess et al., 2009; Brown et al., 2013). Seminal studies by Hubel and Wiesel demonstrated that visual deprivation during the critical developmental period profoundly disrupts V1 binocular processing (Wiesel and Hubel, 1963, 1965; Hubel et al., 1977). Later studies (Kiorpes et al., 1987; Movshon et al., 1987) demonstrated that optical blur can also cause pronounced amblyopia deficits in spatial resolution and contrast sensitivity affecting both V1 function and visual perception. The V1 neuronal deficits tend to be more modest than the visual deficits (Kiorpes et al., 1998; Kiorpes and McKee, 1999; Kiorpes, 2002; Kozma and Kiorpes, 2003; Kiorpes and Movshon, 2004) probably because other extrastriate cortical areas are also affected (e.g., the contrast sensitivity loss is more pronounced for visual perception than V1 neurons; Kiorpes et al., 1998). Consistently, measurements in the prestriate cortex (V2) demonstrate pronounced abnormalities in receptive field structure and orientation tuning that do not appear in V1 and correlate strongly with the visual deficits (Bi et al., 2011; Tao et al., 2014). Amblyopia deficits in ocular dominance balance are also more pronounced in V2 and the middle temporal cortex (MT/V5) than in V1 (El-Shamayleh et al., 2010; Bi et al., 2011), and, in MT, the deficits in motion sensitivity are more pronounced in neuronal populations (Kiorpes et al., 2006) than single neurons (El-Shamayleh et al., 2010).

Amblyopia disrupts visual cortical topography and shape perception through mechanisms that remain poorly understood (Hess, 1982; Levi and Klein, 1986; Sireteanu and Fronius, 1989; Hess et al., 1990; Lagreze and Sireteanu, 1991; Sharma et al., 1999; Barrett et al., 2003). Some distortions in shape perception resemble a simple sum of two gratings (Barrett et al., 2003). However, other distortions are much more complicated and can take the form of zigzag lines, scotomas with irregular geometry, grids with different line thicknesses, and patterns with inhomogeneous local structures. These convoluted distortion patterns would seem to require a complex disruption of local cortical topography. Surprisingly, here we demonstrate that a simple weighted sum of a few rectified sinusoidal gratings can accurately simulate the heterogeneity of pattern distortions reported by human subjects with amblyopia. Moreover, the magnitude of these distortions and the predicted spread of cortical activation are correlated with the severity of amblyopia estimated from deficits in contrast sensitivity. These results support the notion that amblyopia compensates for weakened inputs by increasing the cortical spread of stimulus activation, and the increased spread drives neurons with mismatched orientation preferences that cause perceptual distortions.

## Materials and Methods

We developed a computational model that simulates perceptual distortions of grating patterns drawn by humans with amblyopia (Barrett et al., 2003; sex of human subjects not provided in reference). The model quantifies the magnitude of the perceptual distortions with a new metric that we introduce and that we use to reveal a novel strong correlation between contrast sensitivity deficits and perceptual distortions. We also use a computational model of cortical topography (Najafian et al., 2022) to simulate the cortical spread predicted by perceptual distortions in amblyopia, and we use these simulations to demonstrate a strong correlation between deficits in contrast sensitivity and predicted cortical spread. We also use an animal model to quantify changes in neuronal spatial frequency tuning with contrast loss.

### Model of distorted visual percepts

The distorted visual percepts of human subjects with amblyopia ( $\tilde{P}$) were simulated with a saturated (Sat) and weighted (*W_i_*) sum of cortical filters (*F_i_*), as illustrated in Equation 1. $\tilde{P}$ and *F_i_* are matrices and *W_i_* are scalars. The “x” operator multiplies *W_i_* by each element of *F_i_*, and “Sat” saturates every element of the matrix $\left(\sum_{i=1}^{n}F_{i}\;\times \;W_{i}\right)$:

$$\tilde{P}=\;\mathrm{Sat}\;\left(\overset{n}{\underset{i=1}{\sum }}\;F_{i}\;\times \;W_{i}\right).\;(1)$$

The cortical filters (*F_i_*) were generated by adapting and saturating (Sat) sinusoidal gratings (*G_i_*) with different spatial frequencies and orientations. To simulate different dark/light duty cycles, a constant value (*K_i_*) was added to each sinusoidal grating. This constant value is zero in fellow eyes and ranges from −1 to 1 in amblyopic eyes. The value of *K_i_* is varied to adjust the line thickness of the simulated percepts. Negative values of *K_i_* make dark lines thicker than bright lines and positive values make bright lines thicker than dark lines. The “+” operator adds *K_i_* to each element of *G_i_*, and “Sat” saturates each element of $\left(G_{i}+K_{i}\right)$ matrix with a saturating threshold (*T_i_*; Eq. 2). The values of *T_i_* range from 0 to 2 and are adjusted to optimize the simulations:

$$F_{i}=\;\mathrm{Sat}\;(G_{i}+K_{i})\{\begin{array}{c} F_{i}=\;T_{i},\;if\;(G_{i}+K_{i})\;\geq T_{i}\;\\ F_{i}=-T_{i},\;if\;(G_{i}+K_{i})\leq -T_{i}\\ F_{i}=\;G_{i}+K_{i},\;\mathrm{otherwise},\; \end{array}.\;(2)$$

The weights of the cortical filters (*W_i_*) were computed as the average convolution of the sinusoidal gratings (*G_i_*) and grating stimuli (*S*), scaled by an amblyopia constant (*A_i_*). The values of *A_i_* range from 0 to 1. Increasing *A_i_* makes the *W_i_* stronger and increases the contribution of the respective sinusoidal grating to the simulated percept. *G_i_* and S are matrices and *A_i_* are scalars. The “*” operator convolves the two matrices, S and *G_i_*. The result of $Avg\;(S*G_{i})$ is a scalar value that is multiplied by the scalar *A_i_* (Eq. 3):

$$W_{i}=A_{i}\times \mathrm{Avg}\;(S\;*\;G_{i}).\;(3)$$

### Model optimization

The Nelder–Mead optimization algorithm (Nelder and Mead, 1965) was employed to minimize the perceptual distance between the simulated visual percept ( $\tilde{P}$) and the perceptual drawing (*P*). We used drawings reported in Barrett et al. (2003) from seven amblyopic subjects. All subjects saw the stimuli with the amblyopic eye and saw the drawing with the fellow eye. The stimuli were horizontal, vertical, or oblique sinusoidal gratings (3.2° in diameter) with five different spatial frequencies ranging from 1.25 to 16 cycles per degree (cpd; Barrett et al., 2003). The perceptual distance was quantified using the normalized Laplacian pyramid distance (NLPD) metric described by Laparra et al. (2016) and implemented via the Plenoptic package (Duong et al., 2023; Balzani et al., 2024). This metric measures the dissimilarity across multiple scales in the Laplacian pyramid domain (Eq. 4):

$$\mathrm{NLPD}\;(P,\;\tilde{P})=\;\frac{1}{N}\overset{N}{\underset{k=1}{\sum }}\frac{1}{\sqrt{N_{s}^{\left(k\right)}}}\|y^{\left(k\right)}-{\tilde{y}}_{2}^{\left(k\right)}\|.\;(4)$$

Here, $y^{\left(k\right)}$ and ${\tilde{y}}^{\left(k\right)}$ are vectors representing the transformed original drawing and simulated visual percept at scale *k* in the normalized Laplacian pyramid domain. $N_{s}^{\left(k\right)}$ denotes the number of elements at scale *k*, and *N* is the total number of scales (see Fig. S1 for details).

The simulation starts with a sinusoidal grating (*G_i_*) matched to the stimulus properties and optimizes the model parameters to minimize the perceptual distance (NLPD). If the resulting simulated visual percept is not sufficiently close to the drawing, the model adds an additional sinusoidal grating and optimizes its parameters while keeping the properties of the previous sinusoidal grating fixed. This iterative process continues, adding one sinusoidal grating at a time, until the simulation approximates the drawing within a predefined NLPD threshold. The optimization criteria were adjusted for each individual drawing to minimize the perceptual distance (NLPD) between the drawing and simulated percept as much as possible. The model parameters were also iteratively adjusted until NLPD was minimized and the optimizer converged. Optimization was terminated when both the improvement in NLPD and the maximum change in any model parameter fell below the optimizer tolerance or when the maximum number of iterations was reached. The number of iterations typically ranged from 100 to 400 to achieve the smallest possible NLPD.

### Quantification of visual distortion

To quantify the visual distortion perceived by each amblyopia subject, we selected all stimuli with spatial frequency ≥3 cpd. This selection provided the most robust metric across subjects because visual distortions in amblyopia are most common at high spatial frequencies. We first calculated the absolute value of the difference between the orientation preference of the stimulus (*O_S_*) and the orientation preference of each sinusoidal grating (*O_G_*) contributing to the simulated percept. The contributing sinusoidal grating was classified as matched when $\left|O_{S}-O_{G}\right|$ ≤ 5° and as not-matched when $\left|O_{S}-O_{G}\right|$ > 5°. We then calculated the average weight of the contributing sinusoidal gratings with matched (*W_m_*) and not-matched (*W_n_*) orientation preferences. The magnitude of the visual distortion was then quantified by normalizing $(W_{n}\;-\;W_{m})\times \bar{K}$, where $\bar{K}$ is the average of $\left|K_{i}\right|$ across all sinusoidal gratings and stimuli. *K_i_* is the constant that adjusts the dark/light duty cycle. *W_n_*, *W_m_*, and $\bar{K}$ are all scalars.

### Quantification of contrast sensitivity deficits

To quantify the contrast sensitivity deficits, we fit Gaussian functions (Eq. 5) to the contrast sensitivity functions measured by Barrett et al. for each amblyopic eye and fellow eye (Barrett et al., 2003):

$$CS(sf)=\;g_{0}+\;g\;\;˙e^{-\frac{{\;(sf-sf_{p})}^{2}}{2\sigma^{2}}},\;(5)$$

where *g*_0_ and *g* are the baseline and peak gains at the preferred spatial frequency (*sf_p_*) and σ is the width of the contrast sensitivity function. This Gaussian fit captures the bandpass shape of contrast sensitivity function across spatial frequencies, with the sensitivity decreasing at lower and higher frequencies than the optimal.

Barrett et al. (Barrett et al., 2003) measured contrast sensitivity by asking their subjects to decrease the contrast of the grating stimulus until it was no longer visible. The lowest visible contrast is the contrast threshold, and the contrast sensitivity is 1 divided by the contrast threshold. Barrett et al. (Barrett et al., 2003) reported contrast sensitivities of ∼100 for the lowest spatial frequencies (contrast thresholds ∼1%) and < 10 for the highest spatial frequencies (contrast thresholds > 10%). To quantify contrast sensitivity deficits in amblyopic eyes, we extracted the contrast sensitivity values for each eye plotted by Barrett et al. in their Figure 2 (Barrett et al., 2003). Then, we fitted a Gaussian function to the values measured for each eye and calculated the contrast sensitivity deficit as the fellow/amblyopic ratio of the Gaussian peaks minus one (to keep the range from 0 to infinity across our dataset).

### Simulation of cortical orientation maps

We used a computational model (Najafian et al., 2022) to simulate topographic maps of the macaque primary visual cortex at the center of vision (Blasdel, 1992). The model uses published ocular dominance maps as input (Blasdel, 1992) to simulate a square cortical patch of ∼11 mm² (3.3 × 3.3 mm, 57 × 57 cortical pixels), which is interpolated to generate 228 × 228 cortical pixels with different values of orientation preference and ocular dominance. The average ± standard deviation of the orientation-column width in the macaque cortical patch is 394.9 ± 30.5 microns, and the pinwheel density is 6.1 pinwheels/mm^2^. To facilitate comparisons, we simulated a cortical orientation map for only one eye and then used this map to simulate cortical response maps driven by a sinusoidal grating when the eye was fellow or amblyopic (see quantification of cortical spread below). Using this model, we demonstrate a significant correlation between the predicted cortical spread generated by the grating stimulus in macaque central vision and the visual distortions in human amblyopia (*r* = 0.82; *p* = 0.02). We also demonstrate a significant correlation for a cat model of the primary visual cortex (*r* = 0.87; *p* = 0.01) that uses mosaics of retinal ganglion cells as input (Wässle et al., 1981) instead of the ocular dominance map. In the cat model, we simulated a square cortical patch of ∼25 mm^2^ (5 × 5 mm; 100 × 100 cortical pixels) that received a total of 10,000 thalamic afferents, originating from retinal mosaics made of 50 × 50 retinal ganglion cells of each type (5,000 afferents per eye, 2,500 ON and 2,500 OFF afferents). The average ± standard deviation of the orientation-column width in the cat cortical patch is 546.7 ± 108.3 microns, and the pinwheel density is 3.2 pinwheels/mm^2^.

### Quantification of cortical activation spread

We used the simulations of cortical topography to generate a map of cortical responses driven by stimuli presented to fellow or amblyopic eyes. In the fellow eye, the response of each cortical pixel was determined by just one orientation, the orientation of the stimulus. In the amblyopic eye, the response of each pixel was determined by the multiple orientations of all the filters contributing to the visual percept (weighted according to stimulus orientation similarity). Within our computational framework, perceptual distortions arise because the amblyopic eye drives weaker responses than the fellow eye but within a larger cortical area. Because there is a columnar organization of orientation preference in the primary visual cortex, a column of neurons with similar orientation preference is surrounded by other columns with different orientation preferences. Consequently, the increase in the spread of cortical activation causes the amblyopic eye to pool more neurons from surrounding columns with mismatched orientations than the fellow eye. The stronger responses from the fellow eye may also be more effective at recruiting intracortical inhibition and keeping cortical activity tightly confined to the cortical columns with the same orientation preference as the stimulus. The increased cortical spread for the amblyopic eye is simulated in our model by increasing the orientation mismatches of the neuronal filters that contribute to the visual percepts.

The response strength of each cortical pixel was determined by the difference between the preferred orientation of the cortical pixel and the orientation of the stimulus or amblyopia cortical filters. Our simulation of cortical spread reduces computational load by selecting the most effective stimulus orientations driving visual perception: one orientation when the fellow eye sees one orientation and multiple orientations when the amblyopic eye sees multiple orientation components. Adding a symmetric orientation tuning to the simulations should not affect our quantification of cortical spread if the tuning is similar in normal vision and amblyopia. If the tuning width is increased in amblyopia (Zhu et al., 2022), the cortical spread should be even larger than the one estimated in our simulations.

To simulate cortical responses driven by the fellow eye, we first calculated the orientation mismatch between the cortical pixel (*O_C_*) and stimulus (*O_S_*) measured in degrees, from 0 to 180°. Because 0 and 180° represent the same stimulus orientation, we define a circular distance function (Circ*D*) in Equation 6:

$$\mathrm{Circ}D(O_{C},\;O_{S})=\frac{1}{90}\{\begin{array}{c} \;\;\;|O_{C}-O_{S}|,\;|O_{C}-O_{S}|\leq 90\\ 180-|O_{C}-O_{S}|,\;|O_{C}-O_{S}|>90 \end{array}.\;(6)$$

Here, *O_C_* and *O_S_* are angles in degrees. The factor 1/90 scales the distance so that $\mathrm{Circ}D(O_{C},\;O_{S})$ always lies between 0 and 1. If two orientations differ by 0°, then Circ*D* is equal to 0. If they differ by 90°, then Circ*D* is equal to 1. For differences > 90°, the values are subtracted from 180° (i.e., 180° equals 0° in orientation space). After computing the circular distance, we define the response strength of a cortical pixel with orientation preference *O_C_* to a stimulus with orientation *O_S_*. As the circular distance becomes smaller, the fellow-eye response of the cortical pixel (*FR_C_*) becomes stronger (Eq. 7):

$$FR_{C}=1-\mathrm{Circ}D(O_{C},\;O_{S}).\;(7)$$

Because $\mathrm{Circ}D(O_{C},\;O_{S})$ ranges from 0 (same orientation) to 1 (orthogonal orientations), subtracting the circular distance from 1 yields a response value also between 0 and 1. If *O_C_* is equal to *O_S_*, then CircD is equal to 0, and $FR_{C}$ is equal to 1 (maximum response). Conversely, if *O_C_* is 90° away from *O_S_*, then Circ*D* is equal to 1, and *FR_C_* is equal to 0 (no response).

To simulate cortical responses driven by the amblyopic eye, we calculate the response to all the sinusoidal gratings contributing to the simulated visual percept, with each sinusoidal grating (*G_i_*) having its own orientation (*O_G_*). We apply the circular distance function, replacing *O_S_* with *O_G_* [ $\mathrm{Circ}D(O_{C},\;O_{G})$, Eq. 6], and compute the response of each cortical pixel to each sinusoidal grating, $R_{CG}=1-\mathrm{Circ}D(O_{C},\;O_{G})$. We weigh each sinusoidal grating using the orientation difference between the sinusoidal grating contributing to the simulated percept (*O_G_*) and the stimulus (*O_S_*). We define the grating weight *W_G_* as a Gaussian function centered at zero circular distance from *O_S_* (Eq. 8):

$$W_{G}=\;e^{-\;{\frac{CircD(O_{G},O_{S})}{2\sigma^{2}}}^{2}},\mathrm{with}\;\sigma =0.2.\;(8)$$

When *O_G_* is very close to *O_S_*, then $\mathrm{Circ}D(O_{G},\;O_{S})\approx 0$, and $W_{G}\approx 1$. If the grating orientation is far from *O_S_*, $\mathrm{Circ}D(O_{G},\;O_{S})$ becomes larger, and *W_G_* decreases accordingly. The parameter σ determines the rate at which the weight declines as filter orientations deviate from *O_S_*. We obtain the amblyopic-eye response for each cortical pixel (*AR_C_*) by summing all weighted cortical grating responses (*R_CG_*), where *n* is the number of sinusoidal gratings, as illustrated in Equation 9:

$$AR_{C}=\;\overset{n}{\underset{G=1}{\sum }}\;(R_{CG}\;\times \;W_{G}).\;(9)$$

After the responses from the amblyopic eye (*AR_C_*) and fellow eye (*FR_C_*) are computed for each cortical pixel, we select the cortical pixels that were most strongly activated by stimulus (>70% of the maximum response). We then measure the total cortical spread as the number of strongly activated cortical pixels for amblyopic and fellow (control) eyes. The cortical spread ratio is computed as the amblyopia/control ratio of cortical spread. A ratio equal to one indicates that the cortical spread generated by the stimulus is the same for amblyopic and fellow eyes. A ratio greater than one indicates the cortical spread is larger for the amblyopic eye than for the control eye.

### Electrophysiological recordings

We quantified the effect of contrast loss on neuronal spatial frequency tuning by analyzing electrophysiological recordings from the cat visual thalamus from our laboratory database (collected in 2004; male adult cats, 4–7 kg). All experimental procedures in these animals adhered to the United States Department of Agriculture Animal Welfare Act regulations and were approved by the Institutional Animal Care and Use Committee at the State University of New York, College of Optometry.

The animals were initially anesthetized with ketamine (10 mg kg^−1^, i.m.). Anesthesia was maintained with thiopental sodium (20 mg kg^−1^, i.v.), supplemented as necessary during surgery, and continued during recording at a rate of 1–2 mg kg^−1^ h^−1^, intravenously. Local anesthesia with lidocaine was applied topically or injected subcutaneously at all incision sites and pressure points to ensure adequate analgesia. The animals were intubated and positioned in a stereotaxic apparatus. Throughout the experiment, all vital signs were continuously monitored and maintained within normal physiological ranges. A craniotomy was performed at stereotaxic coordinates (anterior, 5.5 mm; lateral, 10.5 mm) to allow for the insertion of recording electrodes into the LGN. To prevent ocular movements, paralysis was induced with atracurium besylate (0.6–1 mg kg^−1^ h^−1^, i.v.), and artificial ventilation was provided to maintain expired CO₂ levels between 28 and 33 mmHg, ensuring proper respiratory function. Pupils were dilated with atropine sulfate (1%), and nictitating membranes were retracted with Neo-Synephrine (10%). High-permeability contact lenses were placed on the eyes to protect the corneas and achieve appropriate refraction. The optic disc and area centralis were projected onto a tangent screen positioned 114 cm from the animal (Pettigrew et al., 1979).

A circular array of seven independently movable electrodes was introduced into the dorsal LGN (Eckhorn and Thomas, 1993). The electrodes were made of 80 μm quartz/platinum-tungsten fibers sharpened to a fine tip, with impedances ranging from 3 to 6 MΩ (System Eckhorn Thomas Recordings). To minimize interelectrode spacing to ∼80–300 μm, a glass guide tube with an inner diameter of ∼300 μm at the tip was affixed to the shaft of the multielectrode array. The assembly was carefully lowered into the brain, positioning the tip of the guide tube ∼3 mm above the LGN. Each electrode was independently adjusted to target the first main LGN layer (Layer A). The angle of the multielectrode array was precisely calibrated for each experiment, typically set at 25–30° anterior–posterior and 2–5° lateral–central, to ensure simultaneous recording from neurons with spatially overlapping receptive fields. All recordings were conducted within 5–10° of the area centralis. Voltage signals from all seven electrodes were amplified, filtered, and transmitted to a computer running the Discovery software package (Datawave System). Spike waveforms for each neuron were initially identified during the experiment and subsequently verified offline using spike sorting analysis (Plexon Offline Sorter). Visual stimuli were generated via an AT-vista graphics card (Truevision) and presented on a 20 in monitor (Nokia 445Xpro; frame rate, 128 Hz).

### Spatial frequency tuning

To evaluate the spatial frequency tuning of the LGN neurons, we presented full-field sinusoidal drifting gratings at three contrast levels: high (98%), medium (44%), and low (22%). The mean luminance was maintained at 60 cd/m^2^ throughout the experiments. For each contrast level, we measured responses to 10 spatial frequencies, ranging from 0.07 to 6.67 cpd, and we performed 10 trial repetitions per spatial frequency. Each grating stimulus was presented for four cycles at a temporal frequency of 2 Hz. The mean firing rates were measured for each contrast level and spatial frequency and fitted with Gaussian functions to calculate the spatial frequency tuning at different contrasts.

### Receptive field mapping

Spatiotemporal receptive fields were mapped with white noise stimuli made of 16 × 16 white–black checkerboards. Each checkerboard was displayed for 15.5 ms, with individual pixel dimensions subtending 0.9° of the visual angle (Yeh et al., 2003). Receptive fields were measured with reverse correlation by calculating the peristimulus time histogram for each stimulus checks. This approach yielded a three-dimensional array of spatiotemporal responses (*x*-space, *y*-space, time) with a temporal resolution of 15 ms over 285 ms. The responses were then normalized by subtracting the mean and dividing by the maximum absolute value. The resulting receptive fields were further smoothed using two-dimensional interpolation with a scaling factor of 3.

### Experimental design and statistical analysis

Sample sizes were based on prior studies using comparable metrics and datasets (Barrett et al., 2003) and were not determined by a formal power analysis. Data analyses and cortical map simulations were performed using custom MATLAB scripts (versions 2021–2024). The computational model of perceptual distortion and its optimization were implemented in Python (version 3.12.3, Visual Studio Code environment with the following packages: torch 2.3.0 + cpu, torchvision 0.18.0 + cpu, matplotlib 3.9.0, scipy 1.13.1, scikit-image 0.23.2, plenoptic 1.0.2, and numpy 1.26.4). Statistical analyses were conducted in MATLAB (versions 2021–2024) and Python (version 3.12.3). To assess the relationships between continuous variables, we computed Pearson's correlation coefficients. The statistical significance of these correlations was evaluated by calculating the corresponding *p* values. For comparisons between two independent samples, we utilized two-tailed Wilcoxon rank-sum tests. All statistical comparisons using Wilcoxon rank-sum tests are reported in the paper as mean ± standard deviation and *p* values. We applied the binomial test to determine whether the observed ON–OFF dominance distribution significantly deviated from a binomial distribution with specified probabilities. All statistical analyses were performed with the significance level set at *α *= 0.05. We also performed a bootstrap analysis to compare the spatial frequency distributions of stimuli and cortical filters (both calculated with nine spatial frequency bins). For this analysis, we used 1,000 bootstrap samples to measure the average distribution probability of each spatial frequency (random selection of 30 spatial frequencies per sample for both stimuli and filters; uniform random noise with ±2 cpd range added to smooth the distributions).

## Results

We developed a simple computational model that simulates diverse perceptual distortions of grating patterns reported by humans with amblyopia (Barrett et al., 2003). In our model, each distorted visual percept is generated with a weighted sum of sinusoidal gratings, which are rectified before and after the sum to generate cortical filters. Our simple model captures a wide variety of visual distortions very accurately by optimizing just six filter parameters: orientation, phase, spatial frequency, dark/light duty cycle, saturation threshold, and weight. Each parameter makes a unique contribution to the simulated visual percept and is necessary for reproducing the diversity of observed amblyopic distortions. Most simulated distortions have filters with mismatched orientations, as if cortical responses were spreading beyond regions tuned to the preferred orientation of the stimulus. Consistently, our results demonstrate that both the predicted spread of cortical activation and magnitude of visual distortions are correlated with amblyopia severity estimated from deficits in contrast sensitivity. Collectively, these results reveal a possible mechanism that compensates weak responses in the amblyopic cortex by increasing the cortical spread at the cost of generating perceptual distortions.

### Computational model of perceptual distortions in amblyopia

**Figure 1.**
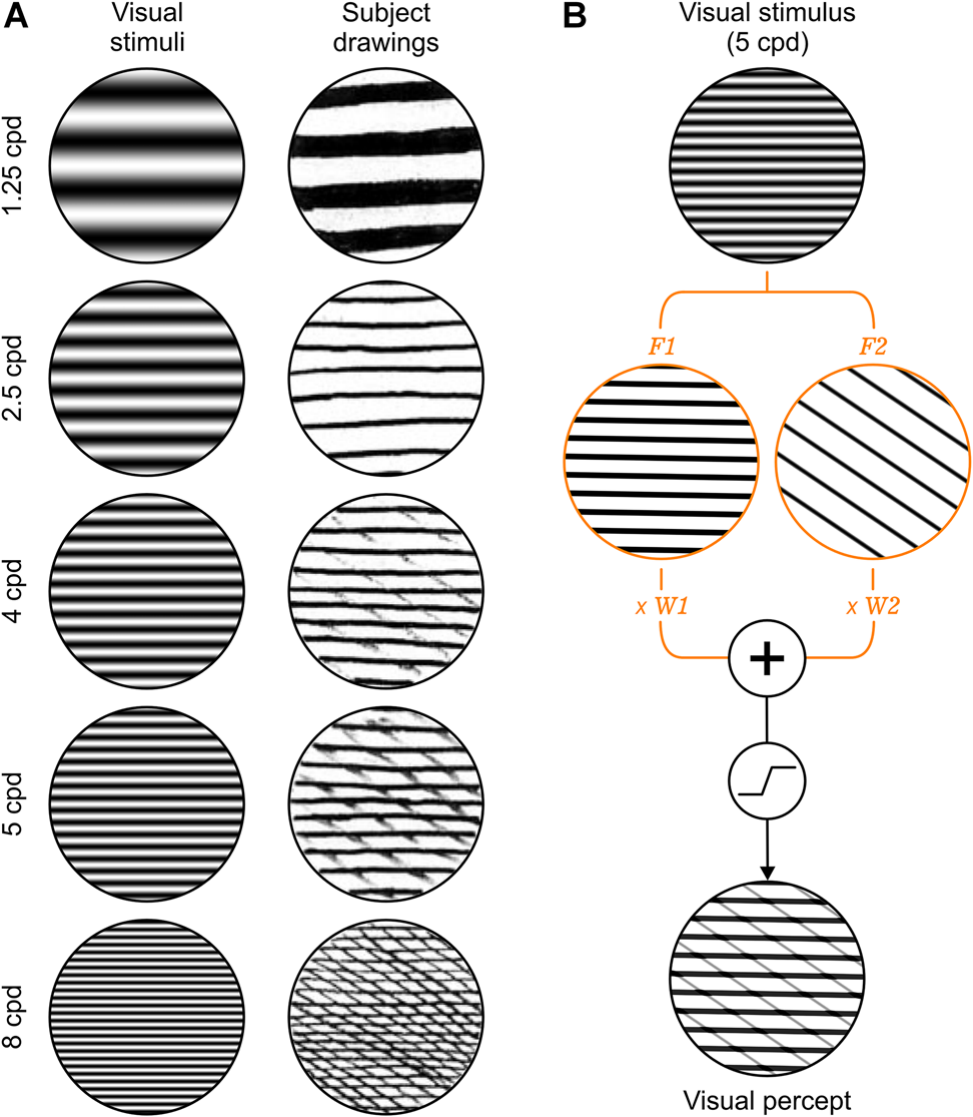
Distorted perception of grating patterns in amblyopia. ***A***, Left column, Horizontal grating stimuli with spatial frequency increasing from top to bottom. Labels on the left, Cycles per degree (cpd). Right column, Subject drawings of the stimuli seen through the amblyopic eye (adapted from Barrett et al., 2003). ***B***, From top to bottom, 5 cpd grating stimulus, two cortical filters generated by the model (*F*_1_ and *F*_2_ in orange), and distorted visual percept simulated by saturating the weighted sum of the filter percepts (weights, *W*_1_ and *W*_2_).

**Figure 2.**
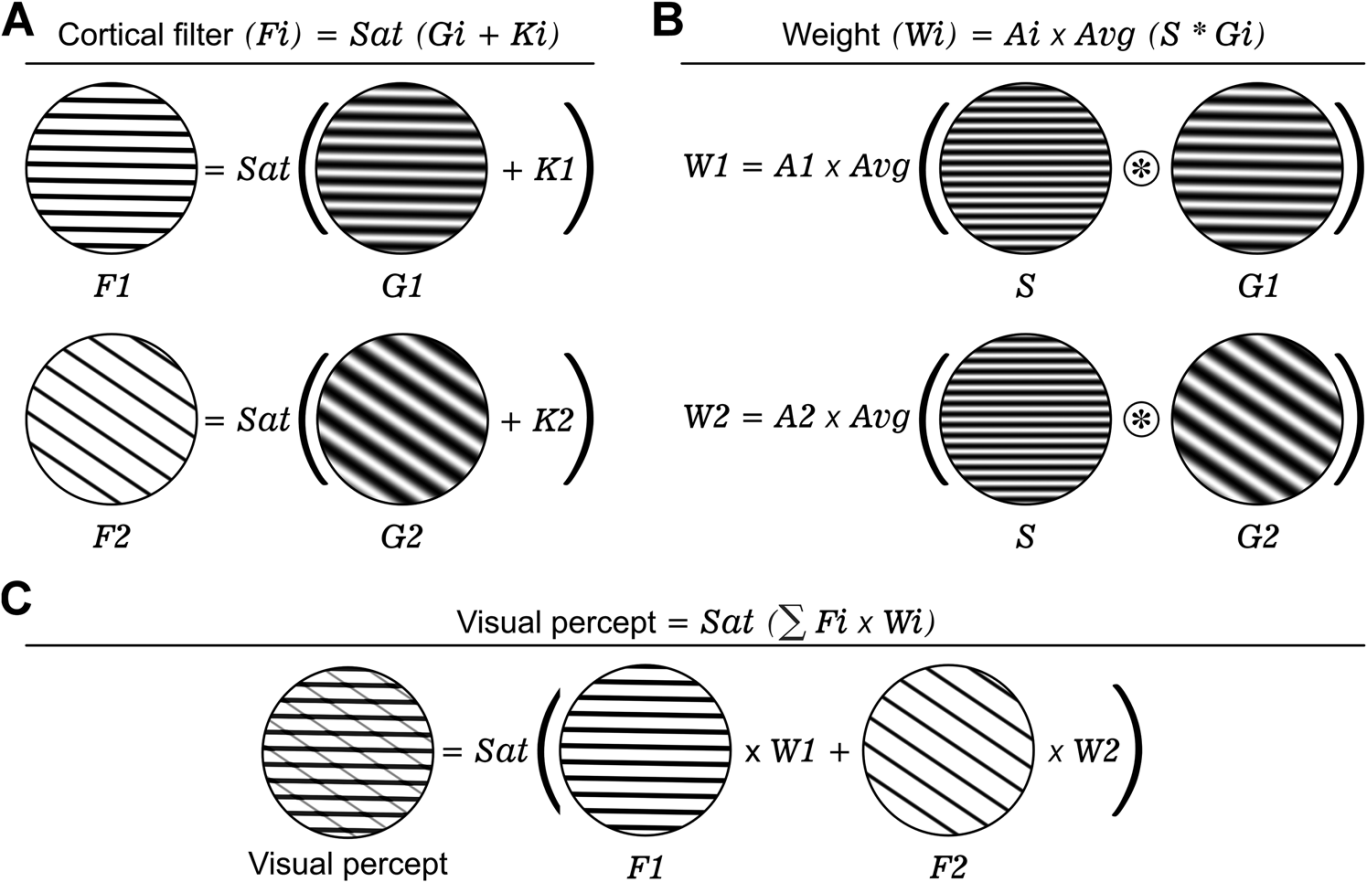
Computational model of distorted visual percepts in amblyopia. ***A***, Each cortical filter (*F_i_*) is obtained by adding a dark/light duty cycle constant (*K_i_*) to a sinusoidal grating (*G_i_*), followed by saturation (Sat). ***B***, The weight (*W_i_*) for each filter is computed as the average of the convolution between the stimulus (*S*) and the sinusoidal grating (*G_i_*), scaled by an amblyopia constant (*A_i_*). ***C***, The final distorted visual percept is generated by saturating the weighted sum of the cortical filters (*F_i_*).

The simplest perceptual distortion in amblyopia is an interference pattern of lines crossing the perceived grating stimulus (Fig. 1*A*). This interference pattern is most visible at high spatial frequencies and has been reported by subjects drawing a grating stimulus seen through the amblyopic eye (Barrett et al., 2003). We have developed a computational model that captures these and more complex perceptual distortions very accurately (Fig. 1*B*). Our model rectifies sinusoidal gratings (*G_i_*) to generate cortical filters (*F_i_*) that are weighted (*W_i_*), summed, and saturated to simulate visual percepts (Fig. 1*B*). Each cortical filter (*F_i_*) is calculated by adding a dark/light constant (*K_i_*) to a sinusoidal grating (*G_i_*), followed by saturation (Fig. 2*A*). Each weight (*W_i_*) is calculated by averaging all the values of the convolution between the stimulus (*S*) and the sinusoidal grating (*G_i_*) and then scaling the result with an amblyopia constant (Fig. 2*B*, *A_i_*; see Materials and Methods for details). The Nelder–Mead algorithm (Nelder and Mead, 1965) is used to optimize the filter parameters based on the perceptual distance between simulated percepts and drawings. The perceptual distance is quantified with the NLPD metric (Laparra et al., 2016), which evaluates dissimilarities across multiple spatial scales (Fig. S1; see Materials and Methods for details). The visual percept is calculated by saturating the weighted sum of multiple cortical filters (Fig. 2*C*). Conceptually, the model simulates the amblyopic activation of neuronal populations with mismatched stimulus preferences and the mismatch causes perceptual distortions.

### Simulations of perceptual distortions in amblyopia

**Figure 3.**
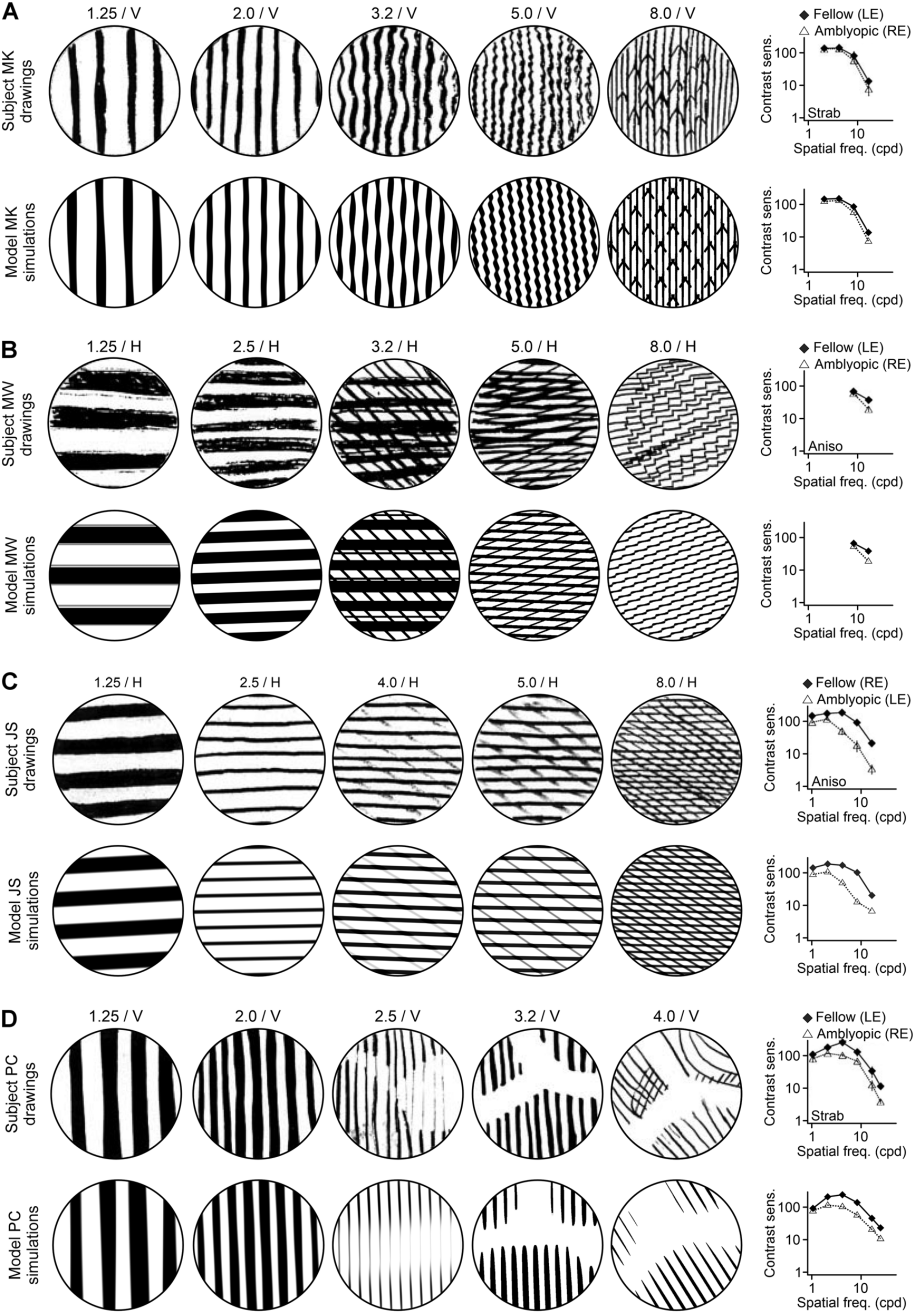
Simulations of distorted visual percepts in four human amblyopes. ***A***, Top row, Drawings of the grating stimuli from subject MK (adapted from Barrett et al., 2003). Labels at the top report the stimulus spatial frequency (cpd) and orientation (H, horizontal; V, vertical). The rightmost panel shows the contrast sensitivity functions for the amblyopic eye (RE, right eye; open symbols) and fellow eye (LE, left eye; filled symbols) and the type of amblyopia (Strab, strabismic; Aniso, anisometropic). Bottom row, Simulated visual percepts and contrast sensitivity functions generated by the model for subject MK. ***B–D***, Same as ***A*** for subjects MW, JS, and PC.

Whereas some perceptual distortions in amblyopia resemble a simple sum of two gratings (Fig. 1), others have more complex patterns of wavy lines, zigzag lines, corners and grids with different line thicknesses, scotomas with irregular shapes, and inhomogeneous local structures (Fig. 3, Fig. S2), which would seem to require a major reorganization of cortical topography. Surprisingly, our simple model reproduces this wide range of perceptual distortions by summing just a few rectified sinusoidal gratings (average, 3.3 filters; Fig. S3).

Our model replicates a large variety of complex distortions that includes distorted wavy lines, patterns of sharp corners (Fig. 3*A*; data from Barrett et al., 2003), distortions in the thickness and spacing of grating bars (Fig. 3*B*; Barrett et al., 2003), and patterns of thin lines increasing in contrast with spatial frequency (Fig. 3*C*; Barrett et al., 2003). Perhaps more surprisingly, our model also replicates Y-shaped scotomas (Fig. 3*D*; see Figs. S2 and S3 for additional examples; notice that the size of the filters is constant and always matches the stimulus size). Across all subjects and stimuli (7 subjects × 5 stimuli = 35 percepts), the average perceptual distance (NLPD) between ideal percepts (stimuli) and drawings was significantly larger than the average perceptual distance between simulated percepts and drawings (1.38  ±  0.27 vs 1.26  ±  0.32; *p* =  0.0002; Wilcoxon test). However, the average perceptual distance between stimuli and drawings was not significantly different than average perceptual distance between stimuli and simulated percepts (1.38  ±  0.27 vs 1.39  ±  0.31; *p* =  0.78; Wilcoxon test). Therefore, replacing drawings with simulated percepts reduced the average perceptual distance to the stimuli by only 0.14%. However, replacing the stimuli with simulated percepts reduced the average perceptual distance to the drawings by 9.03% (a reduction more than one order of magnitude larger). We use our model to quantify the magnitude of the perceptual distortions and compare our new metric of distortion magnitude with contrast sensitivity deficits (Fig. 3, right panels) measured by Barrett et al. (Barrett et al., 2003).

### Contrast modulations of neuronal spatial frequency tuning

**Figure 4.**
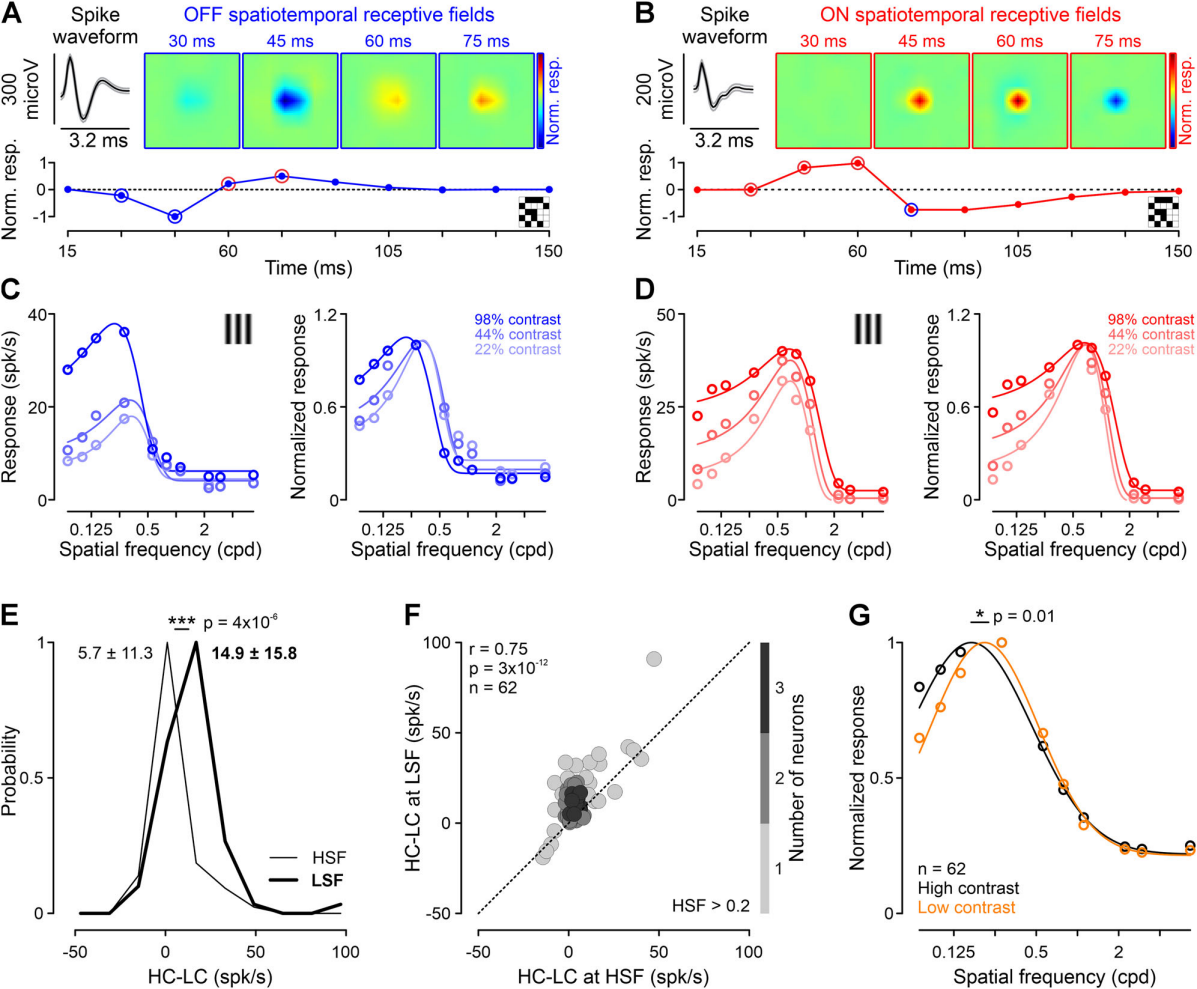
Contrast modulations of neuronal spatial frequency tuning. ***A,B***, Top row, Spike waveform (average ± standard deviation) and spatiotemporal receptive field of single LGN neurons (blue, OFF response to dark stimuli; red, ON response to light stimuli). Bottom row, Normalized temporal impulse response and icon of the white noise stimulus used to measure receptive fields. ***C,D***, Spatial frequency tuning of the same LGN neurons measured with three contrasts (color-coded with different color saturation). Icon illustrates the grating stimulus. Left, Response in spk/sec. Right, Normalized response. Circles are data points and lines are fitted curves. ***E***, Response difference between high-contrast (HC) and low-contrast (LC) stimuli, measured at low (LSF, ≤0.2 cpd; thick line) and high spatial frequencies (HSF, >0.2 cpd; thin line). Response average ± standard deviation shown at the top-left for HSF and top-right for LSF (*p* = 4 × 10^−6^; Wilcoxon test comparing responses to HSF and LSF). ***F***, Correlation between HSF and LSF of HC–LC response difference. The dashed line represents the unity line, and grayscale intensity represents neuron density. ***G***, Averaged spatial frequency tuning for two grating contrasts (high, 98%; black; low, 22%; orange; lines are Gaussian fits). The preferred spatial frequencies for the two contrasts are significantly different (*n* = 62; *p* = 0.01; Wilcoxon test).

Amblyopia causes a loss of contrast sensitivity that could potentially affect the neuronal spatial frequency tuning and distort the spatial–frequency components of visual percepts. This distortion could explain why our simulated perceptual distortions often require using filters with mismatched spatial frequencies (Fig. S3). To test this hypothesis, we analyzed the spatial frequency tuning of neurons measured with different stimulus contrasts in the cat dorsal LGN. LGN neurons were classified as OFF (Fig. 4*A*) or ON center (Fig. 4*B*) by mapping their receptive fields. We also measured their spatial frequency tuning with drifting gratings of different contrasts (Fig. 4*C*,*D*). This analysis demonstrates that reducing stimulus contrast makes visual responses weaker in both OFF (Fig. 4*C*) and ON pathways (Fig. 4*D*), but the response reduction is not the same across spatial frequencies. In our neuronal population with an average preferred spatial frequency of 0.2 cpd (0.2 ± 0.12; *n* = 62 with *R*^2^ goodness of fit for spatial frequency tuning > 0.9), low contrast reduced visual responses two times more for ≤0.2 cpd than >0.2 cpd stimuli (Fig. 4*E,F*; 14.9 ± 15.8 vs 5.7 ± 11.3; *p* = 4 × 10^−6^; Wilcoxon test). This larger response reduction at low spatial frequencies caused a paradoxical increase in preferred spatial frequency, which could be demonstrated in the average of all LGN neurons (Fig. 4*G*; 0.2 ± 0.12 cpd vs 0.23 ± 0.11 cpd; *p* =  0.01; *n* = 62; Wilcoxon test) and OFF LGN neurons (0.18 ± 0.08 vs 0.23 ± 0.09 cpd; *p* = 0.003; *n* = 41; Wilcoxon test), but not in ON LGN neurons (0.22 ± 0.17 vs 0.23 ± 0.13 cpd; *p* = 0.79; *n* = 21; Wilcoxon test), probably because ON neurons saturate more with contrast (Kremkow et al., 2014; Pons et al., 2019; Poudel et al., 2024).

Consistent with previous studies in the primary visual cortex of both cats and macaques (Skottun et al., 1986; Skottun et al., 1987; Sceniak et al., 2002), our results demonstrate that contrast affects the spatial frequency tuning of individual neurons. However, unlike these previous studies, the neurons in our sample had low optimal spatial frequencies and reduced their responses mostly to spatial frequencies lower than the optimal. There is some evidence that the effect of contrast may depend on the optimal spatial frequency of the neuron (Skottun et al., 1986). Therefore, taken together with previous studies, our measurements suggest that contrast reduces spatial frequencies lower than the optimal in neurons tuned to low spatial frequencies and higher than the optimal in neurons tuned to high spatial frequencies.

**Figure 5.**
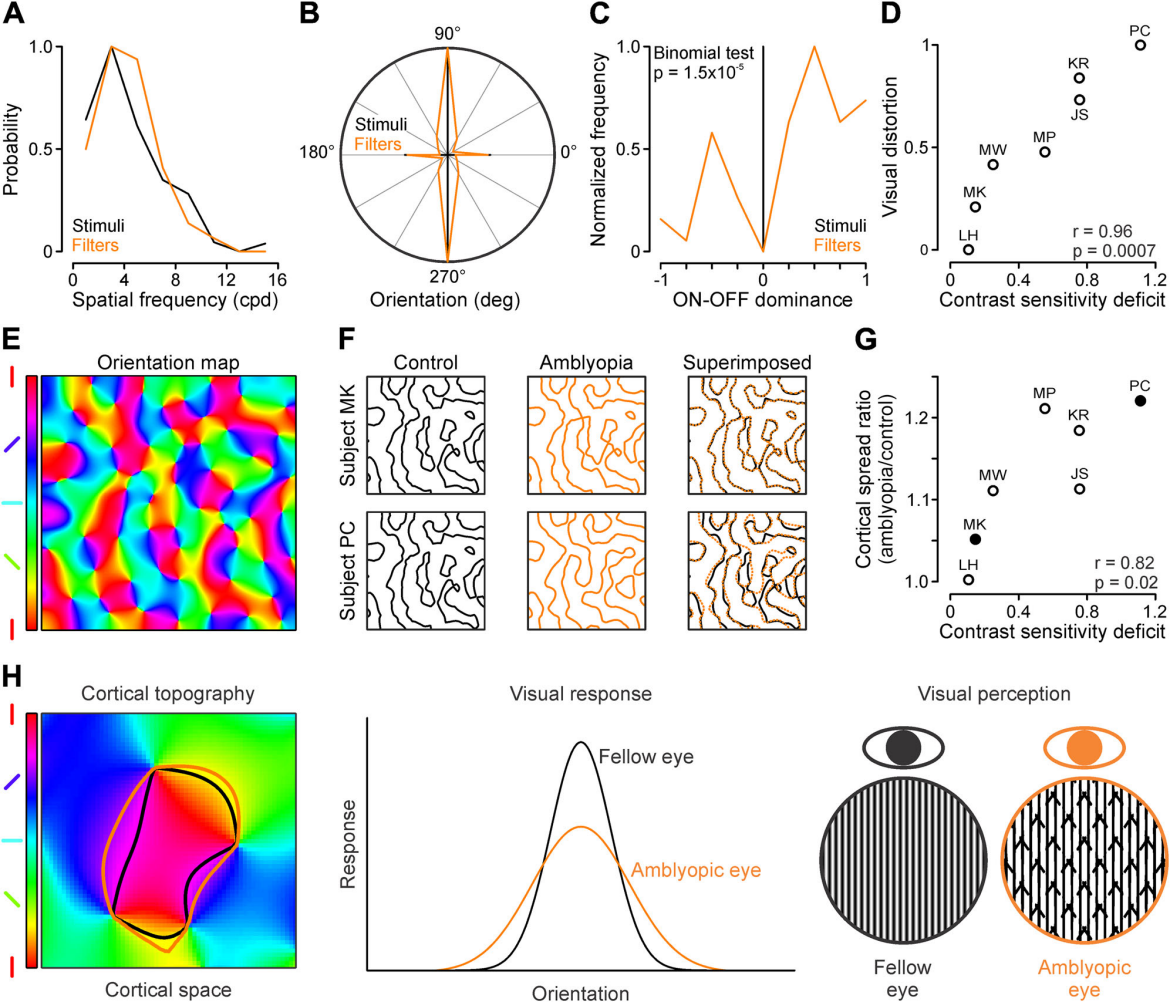
Visual distortions increase with contrast sensitivity deficits and predicted cortical spread. ***A***, Spatial frequency distributions of stimuli (black) and gratings from simulated cortical filters (orange), obtained with 1,000 bootstrap samples (30 spatial frequencies per sample). ***B***, Normalized orientation distributions of stimuli (black) and gratings from simulated cortical filters (orange). ***C***, Normalized distribution of ON–OFF dominance (dark/light duty cycle) for stimuli (black) and gratings from simulated cortical filters (orange). ***D***, Deficits in contrast sensitivity are correlated with severity of visual distortions in amblyopia (initials above/below symbols). ***E***, Simulated cortical orientation map for one eye with color-coded orientation preference. ***F***, From left to right, Simulated cortical patterns generated by a vertical grating (contour lines, 70% of maximum response) for the control eye (black), amblyopic eye (orange), and superimposed eyes in two example subjects (top, MK; bottom, PC). ***G***, Deficits in contrast sensitivity are correlated with the predicted cortical spread in amblyopia (initials above/below symbols). Filled symbols correspond to the subjects highlighted in panel ***F***. ***H***, From left to right, a patch of cortical orientation map overlaid with region contours activated by vertical gratings in control (black) and amblyopic (orange) eyes, a schematic illustrating the population orientation tuning activated by the two eyes, and the visual percepts (fellow, black; amblyopic, orange).

As with thalamic neurons, the filters used to simulate amblyopia perceptual distortions were frequently mismatched in spatial frequency (Figs. S3 and S4). Moreover, consistently with the larger distortions at high spatial frequencies, the number of filters required to simulate perceptual distortions was positively correlated with the spatial frequency of the stimulus (*r* = 0.39; *p* = 0.02). The average spatial frequency was also lower in the cortical filters than the original stimuli, but the difference was not significant (4.7 ± 3.4 vs 3.7 ± 2.2 cpd; *n* = 102 pairs; *p* = 0.14; Wilcoxon test), probably because some filters had higher spatial frequency than the stimulus [Fig. S4; see also Fig. 7 of Barrett et al. (Barrett et al., 2003)]. Consistent with this interpretation, a bootstrapped analysis of the filters spatial frequency revealed a small paradoxical shift toward higher frequencies (Fig. 5*A*) similar to the shift caused by contrast reduction in thalamic neurons (Fig. 4*G*). It should be noted that these spatial frequency mismatches are relatively small and can be influenced by the drawing accuracy of the subjects (Fig. S4). By comparison, the mismatches in filter orientation (Fig. 5*B*) and dark/light duty cycle (Fig. 5*C*, Fig. S4) were much more pronounced and are likely to contribute more to the perceptual distortions.

### Magnitude of visual distortions correlates with contrast sensitivity deficits in amblyopia

If amblyopia disrupts both shape and contrast perception, the magnitude of the amblyopia visual distortions should be correlated with the deficits in contrast sensitivity. To test this hypothesis, we quantified the distortion magnitude of gratings with spatial frequency ≥3 cpd, which are most frequently distorted in amblyopia. The distortion magnitude was quantified as the absolute difference between the average weight of cortical filters with orientations matched and not-matched to the stimulus orientation (matched average–not-matched average; matched, ≤5° orientation difference; mismatched, >5° orientation difference). This average difference was then multiplied by the absolute dark/light (*K*_i_) average of the cortical filters and normalized to a range between zero and one. We also quantified the deficit in contrast sensitivity as the peak spatial–frequency ratio between fellow and amblyopic eyes minus one (to have a range from 0 to infinity). This analysis revealed a novel and important finding. The magnitude of visual distortions in amblyopia is strongly correlated with the contrast sensitivity deficit (*r* =  0.96; *p* =  0.0007; Fig. 5*D*). Greater sensitivity deficits are associated with greater visual distortions.

In the visual cortex, a reduction in stimulus contrast weakens neuronal responses while increasing cortical spread (Nauhaus et al., 2009; Swadlow and Alonso, 2009). Therefore, the contrast sensitivity loss and response weakening in amblyopia could lead to a similar increase in cortical spread. To test this hypothesis, we simulated monocular cortical responses to grating stimuli (Fig. 5*E*) using a computational model of the macaque primary visual cortex at central vision (Najafian et al., 2022), instructed by experimental measurements (Blasdel, 1992). The simulations for the fellow eye used the original stimuli as inputs, while the simulations for the amblyopic eye used the perceptual filters contributing to the visual distortions (Fig. 5*F*; see Materials and Methods for details). For both eyes, we used the grating orientations (vertical or horizontal) and spatial frequencies (4 or 5 cpd) that generated the most pronounced perceptual distortions in each subject. The computational model demonstrates that simulated cortical areas generating strong responses (≥70% of maximum) are 17.16 ± 11.55% larger for the amblyopic than the fellow eye (Fig. 5*F*).

More importantly, the contrast sensitivity deficit in amblyopia was strongly correlated with the amblyopic/fellow-eye ratio of cortical activation spread (*r* =  0.87; *p* =  0.01; Fig. 5*G*). Thus, as contrast sensitivity decreases, the predicted spread of cortical activation becomes larger. This simple simulation reveals the expected larger cortical spread for distorted perceptions that have multiple orientation components. Taken together, our results reveal a link among contrast sensitivity deficits, predicted spread of cortical activation, and magnitude of perceptual distortions in amblyopia. They also suggest that weak inputs in the amblyopic cortex are compensated by an increase in cortical spread that resembles the effect of low contrast in the visual cortex (Nauhaus et al., 2009; Swadlow and Alonso, 2009). According to this mechanism, the increase in cortical spread activates neurons with mismatched stimulus preferences that generate perceptual distortions (Fig. 5*H*). Therefore, weak inputs in amblyopia explain both deficits in contrast sensitivity and distortions in shape perception.

## Discussion

We introduce a simple computational model that simulates perceptual distortions of grating patterns reported by humans with amblyopia (Barrett et al., 2003). The model uses an average of ∼3 filters to reproduce a large variety of complex visual distortions that include patterns of wavy or zigzag lines, corner patterns, grids with different line thicknesses, and scotomas with irregular shapes. We use the model to quantify the magnitude of the visual distortions and use this quantification to reveal strong correlations among the magnitude of visual distortions, contrast sensitivity deficits, and predicted spread of cortical activation. We also use our findings to propose a neuronal mechanism that compensates weak visual responses in amblyopia by increasing cortical response spread, a compensatory mechanism that resembles the effects of lowering stimulus contrast in the visual cortex (Nauhaus et al., 2009; Swadlow and Alonso, 2009). In this mechanism, the increased spread of cortical activation drives neurons with mismatched stimulus preferences that generate visual distortions.

The diverse visual distortions reported in amblyopia (Hess et al., 1978; Bradley and Freeman, 1985; Thibos and Bradley, 1993; Barrett et al., 2003) posed significant challenges to previous models of cortical function (Hess, 1982; Levi and Klein, 1986; Hess et al., 1990; Barrett et al., 2003). Some simple distortions resembling interference patterns could be explained by summing two sinusoidal gratings with different orientations, spatial frequencies, and contrast, but other more complex distortions such as corner patterns or scotomas with irregular shapes could not be explained (Barrett et al., 2003). By adding three parameters (rectification, phase, and dark/light duty cycle) and increasing the number of filters, our simple model extends the pioneering work from Barrett et al. (2003) in several important new directions. First, the model simulates all major amblyopia distortions of grating patterns, and the simulations are more accurate than in the past. Second, the model provides a new metric to quantify visual distortions, and the new metric is strongly correlated with amblyopia deficits in contrast sensitivity. Third, the model simulations provide support for a cortical mechanism that compensates weak visual responses by increasing the cortical response spread. Fourth, the finding that a large variety of visual distortions can be simulated with the sum of just a few grating filters is surprising and demonstrates that major rearrangements in cortical topography are not needed to explain amblyopia visual distortions.

Complex perceptual distortions in amblyopia have been thought to originate from severe disruptions of cortical retinotopy, either through uncalibrated neural disarrays (Hess, 1982; Hess et al., 1990) or retinotopic undersampling (Levi and Klein, 1986). However, random disruptions of cortical topography cannot explain why some patterns of amblyopia distortions are more common than others and can simulate only simple distortions (Sharma et al., 1999). Our model provides a possible explanation for the surprising diversity of visual distortions in amblyopia: an increase in cortical spread. The larger cortical spread should increase the neuronal scatter of orientation preference, phase, and retinotopy contributing to the visual percept. In turn, the increased scatter should distort not only grating patterns but also the position, shape, and orientation of local stimuli such as letters (Sayim and Cavanagh, 2013; Sayim and Wagemans, 2017; Melnik et al., 2021). In the mechanism that we propose, amblyopia weakens visual responses while increasing cortical spread, replicating the effect of contrast reduction in the visual cortex (Nauhaus et al., 2009; Swadlow and Alonso, 2009). The weak responses reduce contrast sensitivity by decreasing the number of activated neurons, just as contrast sensitivity decreases when the volume of the primary visual cortex is reduced in normal subjects (Himmelberg et al., 2022). The weak cortical responses also increase the cortical spread (Nauhaus et al., 2009) as if the cortex were stimulated with low spatial frequencies (Kremkow et al., 2014), making amblyopia central vision similar to normal peripheral vision, as proposed by Levi and Klein (1985). This simple mechanism provides a unified explanation that integrates principles of cortical physiology/topography with contrast/shape perception. It also captures the heterogeneity of amblyopic visual distortions without requiring an extensive reorganization of cortical topography, which has been notoriously difficult to demonstrate (Horton and Stryker, 1993). The mechanism is also consistent with increased crowding through abnormal pooling of visual information in amblyopia (Levi et al., 2002).

We notice that, whereas visual deprivation causes a pronounced shrinkage of ocular dominance columns in the primary visual cortex (Wiesel and Hubel, 1963, 1965; Hubel et al., 1977), the shrinkage has not been found in moderate amblyopia (Horton and Stryker, 1993; Nasr et al., 2025). Our results suggest that moderate amblyopia may increase cortical spread by weakening intracortical inhibition (Hensch and Stryker, 2004), whereas visual deprivation may reduce overall activity much more strongly, causing a structural shrinkage of ocular dominance columns.
